# Integrative sWGS: A New Paradigm for HRD Detection in Ovarian Cancer

**DOI:** 10.3390/ijms262411968

**Published:** 2025-12-12

**Authors:** Dan Corneliu Jinga, Georgiana Duta-Cornescu, Danut Cimponeriu, Eirini Papadopoulou, Angeliki Meintani, George Tsaousis, Amalia Chirnogea, Irina Bucatariu, Polixenia-Georgeta Iorga, Diana Chetroiu, Sorin-Cornel Hosu, Amalia Hogea-Zah, Mircea-Dragos Median, Bogdan Diana, Dana-Lucia Stănculeanu, Raluca Mihaila, Dana-Sonia Nagy, Pompilia-Elena Motatu, Turcanu Eugeniu, Elena-Octaviana Cristea, Ion-Cristian Iaciu, Paul Kubelac, Andreea Truican

**Affiliations:** 1 Neolife Medical Center, 013975 Bucharest, Romania; danjinga2002@yahoo.com; 2 Department of Medical-Surgical and Preventive Disciplines, Faculty of Medicine, Titu Maiorescu University, 040051 Bucharest, Romania; 3 Department of Applied Genetic and Biotechnology, Faculty of Biology, University of Bucharest, 050095 Bucharest, Romania; georgiana.duta-cornescu@bio.unibuc.ro (G.D.-C.); danut.cimponeriu@bio.unibuc.ro (D.C.); 4 Genekor Medical S.A., 153044 Athens, Greece; eirinipapad@genekor.com (E.P.);; 5 Genekor Medical SRL, 014145 Bucharest, Romania; amalia.chirnogea@genekor.com (A.C.);; 6 General Oncology Unit, University Emergency Hospital Bucharest, 050098 Bucharest, Romania; polixeniaiorga@yahoo.com (P.-G.I.);; 7 General Oncology Unit, Alba Iulia Country Hospital Emergency, 510007 Alba Iulia, Romania; 8 General Oncology Unit, Satu Mare Country Emergency Hospital, 440192 Satu Mare, Romania; 9 General Oncology Unit, Filantropia Clinical Hospital, 011171 Bucharest, Romania; dragos.median@gmail.com; 10 Donna Medical Center, 021755 Bucharest, Romania; 11 Institute of Oncology Prof. Dr. Alexandru Trestioreanu, 022328 Bucharest, Romania; 12 Oncohelp Oncology Center, 300239 Timisoara, Romania; 13 General Oncology Unit, Ploiești Municipal Hospital, 100337 Ploiesti, Romania; 14 Neolife Medical Center, 700503 Iasi, Romania; 15 General Oncology Unit, Elias University Emergency Hospital, 011461 Bucharest, Romania; 16 Institute of Oncology Prof. Dr. I. Chiricută, 400015 Cluj-Napoca, Romania; 17 Faculty of Medical and Health Sciences, Babeș-Bolyai University, 9 Clinicilor Street, 400347 Cluj-Napoca, Romania

**Keywords:** sWGS, genomic instability, homologous recombination deficiency, PARP inhibitors, ovarian cancer

## Abstract

Homologous recombination deficiency (HRD) is a clinically relevant biomarker that predicts sensitivity to PARP inhibitors and enables personalized cancer therapy. Validated local HRD testing solutions are essential to ensure timely and equitable access, ultimately improving treatment outcomes. We evaluated a shallow whole-genome sequencing (sWGS) approach for genomic instability (GI) assessment combined with a 52-gene targeted panel in ovarian cancer. Validation used reference materials and 24 archival samples with prior HRD characterization, comparing performance with the Myriad myChoice^®^ HRD test. A prospective cohort of 124 newly diagnosed ovarian cancer patients was then analyzed. sWGS-derived GI status showed strong concordance with the reference test (95.8% overall agreement; κ = 0.913; NPV 100%, PPV 93.3%). Pathogenic *BRCA1/2* variants were detected in 30 patients (24.19%). An additional 22.76% were *BRCA1/2*-negative but GI-positive, giving an overall HRD prevalence of 47.15%. Platinum sensitivity occurred in 90.0% (18/20) of HRD-positive patients with follow-up. Among 12 patients assessed for PARP-inhibitor response, the overall response rate was 66.7% (95% CI 39.1–86.2) and disease control rate 83.3% (95% CI 55.2–95.3). *TP53* alterations were most frequent (62.90%), followed by *BRCA1* (19.35%) and *BRCA2* (4.83%). Pathogenic variants in other HR-pathway genes (*ATM, CHEK2, BRIP1, RAD51C, BARD1*) appeared in 9.57% of BRCA-wild-type cases, with heterogeneous GI impact. Two cases showed concurrent *BRCA2* variants and microsatellite instability, indicating possible eligibility for anti-PD-1/PD-L1 therapy in addition to PARPi. This first comprehensive analysis of Romanian ovarian cancer patients suggests that integrating sWGS-based genomic instability assessment with BRCA testing can improve HRD detection and reflects the heterogeneity of HR-pathway variants. Preliminary clinical observations were consistent with known HRD-associated treatment responses, although larger studies are needed to confirm these findings.

## 1. Introduction

Ovarian cancer remains the leading cause of mortality among gynecologic malignancies, despite improvements in survival rates due to targeted treatment. Most patients experience a recurrence after an initial response to platinum-based chemotherapy, and the long-term prognosis remains poor [1]. Various factors may affect tumor progression and resistance to platinum-based chemotherapy, including alterations in tumor cells, the tumor’s biophysical characteristics, and the tumor microenvironment [1,2,3]. Furthermore, recent advancements in translational research, including patient-derived xenograft (PDX) and organoid models, have underscored the considerable molecular heterogeneity of ovarian cancer and its influence on therapeutic response [4]. Consequently, the development of dependable biomarkers and innovative therapeutic approaches is crucial for refining treatment selection and improving survival outcomes.

Among the molecular mechanisms that affect treatment response in ovarian cancer, deficiencies in the homologous recombination (HR) DNA repair pathway constitute one of the most clinically significant factors. The HR DNA repair pathway is employed during DNA replication for error-free repair of the double-strand breaks. A deficiency in this machinery results in an accumulation of double-strand breaks and genomic instability, hence driving carcinogenesis. Homologous recombination deficiency (HRD) is a biological characteristic observed in more than fifty percent of ovarian tumors, occurring when the HR process is unable to efficiently repair DNA double-strand breaks [5].

HRD most often arises from pathogenic variants in *BRCA1* or *BRCA2* but can also be caused by defects in repair pathway genes or by epigenetic changes that silence HR function. Its clinical importance has grown in recent years with the introduction of poly (ADP-ribose) polymerase inhibitors (PARPi), which exploit HRD through synthetic lethality. Multiple randomized trials have shown that PARPi improve outcomes in ovarian cancer patients not only in *BRCA*-mutated cancers but also in a subset of *BRCA*-wildtype tumors with high genomic instability [5,6,7].

Currently PARP inhibitors have been approved apart for ovarian cancer also for breast and pancreatic cancer patients harboring germline *BRCA1/2* alterations as well as in prostate cancer patients with *BRCA1/2* and other HR gene alterations, both germline and somatic. Since PARPi sensitivity is enhanced in biomarker positive patients, accurate biomarker testing is imperative for appropriate patient selection. The aim is to identify those patients most likely to respond, while maintaining high levels of both sensitivity and specificity [8].

The Myriad myChoice^®^ HRD test is a widely used diagnostic tool that integrates *BRCA1/2* analysis with genomic instability-related genomic scar analysis, derived from genome-wide measures of chromosomal change [9]. The Genomic Instability Score (GIS) is a metric that quantifies the combined impact of three critical genomic scar signatures: Loss of Heterozygosity (LOH), Large-Scale Transitions (LST), and Telomeric Allelic Imbalance (TAI) [9].

The presence of either *BRCA1/2* alteration or a GIS > 42 defines a tumor as HRD positive. Although this assay has been clinically validated, it is centralized, has a relatively high cost, and may not be easily accessible in all healthcare settings. These limitations have driven interest in alternative approaches that can be implemented locally without compromising accuracy [9,10].

Therefore, several assays have emerged to assess genomic scars. Most of them are based on NGS for the analysis of Genomic Instability, while also identifying pathogenic variants in other genes involved in the HR pathway [10,11,12,13].

However, differences in methodology utilized, scoring algorithms, and cutoff thresholds of the various HRD testing platforms may lead to significant degree of variability, which could impact the interpretation and comparability of results [9]. Consequently, there are currently ongoing international initiatives to standardize HRD assays to facilitate comparisons between various clinical settings and studies. Standardization is essential, as discrepancies in assay design can significantly impact patient classification and, as a result, therapeutic decisions regarding the eligibility of PARP inhibitors. The reliability of results in a clinical setting is contingent upon the validation of methodologies, which is imperative due to the variability of current HRD assays [14,15]. Among the approaches used, low-coverage whole-genome sequencing of tumor DNA combined with bioinformatic models that integrate multiple measures of genomic instability can provide a GI measurement comparable to those reported by established commercial platforms [13,16].

While HRD testing has been increasingly adopted in international clinical practice, data on its implementation and genomic landscape in ovarian cancer patients from Romania are lacking [17]. This highlights the necessity of conducting region-specific genomic analyses to guide evidence-based treatment strategies and align local practices with international standards.

In the present study, we examined the performance and applicability of a Shallow whole-genome sequencing (sWGS) for GI calculation combined with a 52 gene panel for HR gene analysis in tumor tissue. The new sWGS-based genomic instability assay was validated by comparing its performance to the reference test for HRD detection, Myriad myChoice^®^, in ovarian cancer samples as well as reference materials.

## 2. Results

Reference material DNA analysis revealed a high concordance between expected and calculated GIS results. The values obtained were as follows: for the 2 high positive reference samples the GIS values were 0.99 and 0.98 (expected 72 ± 2); for the 2 low positive reference materials the values were 0.91 and 0.94 (expected 54 ± 2) and for the 2 negative reference materials the GIS values were 0.28 and 0.30 (expected 31 ± 2).

A total of 24 tumor samples were analyzed using the newly developed sWGS GI assay and compared against the Myriad GIS reference test. The classification into high or low GI status showed high agreement between the two tests (Table 1). The overall percent agreement (OPA) was 95.83% (23/24), with a positive predictive value (PPV) of 93.33% (14/15) and a negative predictive value (NPV) of 100% (9/9). Sensitivity was 100% (14/14) and specificity was 90.00% (9/10).

**Figure 1:**
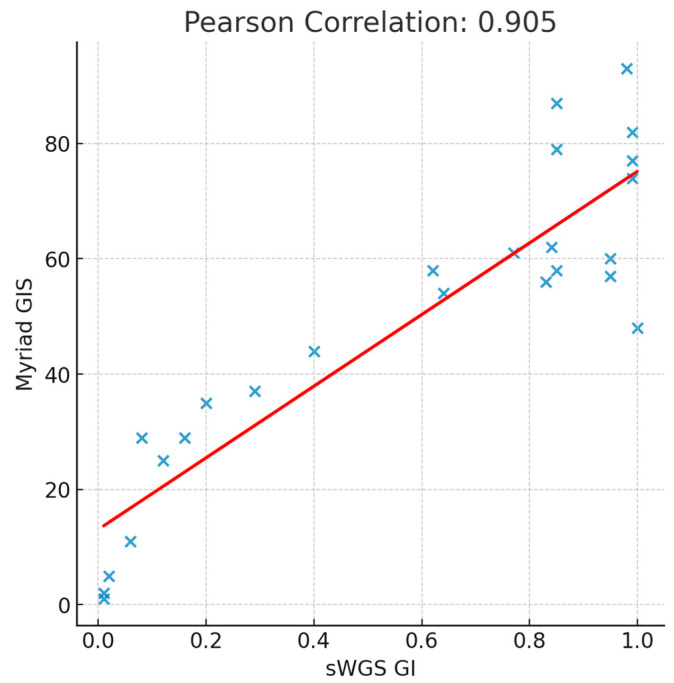
Pearson correlation between the reference test (Myriad) and shallow Whole Genome Sequencing (sWGS) in Genomic Instability (GI) status determination.

Cohen’s kappa (κ) coefficient for agreement in GI classification was 0.913, indicating almost perfect agreement between the assays. Comparison of continuous GI scores showed a Pearson correlation coefficient of 0.905 and a Spearman correlation coefficient of 0.844, confirming a strong linear and monotonic relationship. The scatter plot in Figure 1 illustrates this relationship, with values tightly aligned to the regression line. These results demonstrate that the sWGS GI assay yields result highly consistent with the established Myriad GIS test, supporting its potential as a robust and reliable alternative for genomic instability assessment in HRD testing for metastatic ovarian cancer.

### 2.1. HRD Results in the Ovarian Cancer Cohort

Histopathological assessment revealed that high-grade serous ovarian carcinoma (HGSC) represented the predominant subtype, observed in 106 of 124 patients (85.48%). Non-serous subtypes were less frequent and included clear cell (n = 5), endometrioid (n = 4), mucinous (n = 2), low-grade serous (n = 1), seromucinous (n = 1), and borderline serous tumors (n = 2), while the histologic subtype was not specified in 3 patients. One sample failed GI quality control and was excluded, leaving 123 evaluable cases for GI evaluation. Pathogenic or likely pathogenic *BRCA1/2* variants were detected in 30 of the 124 patients (24.19%). Median age at diagnosis was 61.1 years (range 38–73) for *BRCA*-positive patients and 65.1 years (range 34–84) for *BRCA*-negative patients; the difference did not reach statistical significance (*p* = 0.116). Moreover, no statistically significant association was observed between age at diagnosis and genomic instability (GI) status (median 65.3 years for GI-positive vs. 64.4 years for GI-negative, *p* = 0.451).

Of the 94 patients who tested negative for *BRCA1/2* alterations, successful GI assessment was possible in 93 cases (98.94%). Of those, 28 patients (30.11%) were classified in the category of GI positive status, using the probability threshold of SeqOne > 0.5. As would be expected, the GI-high category was exclusively confined to the high-grade serous carcinomas (28/28), whereas non-serous subtypes showed only GI-low cases, consistent with their distinct molecular profiles. However, given the limited sample size and low incidence of certain histological subtypes, the available data are insufficient to support definitive conclusions. Additionally, *BRCA1/2* mutations and GI-high status were largely confined to FIGO stage III–IV tumors, but the low number of early-stage cases precluded meaningful statistical testing.

**Figure 2:**
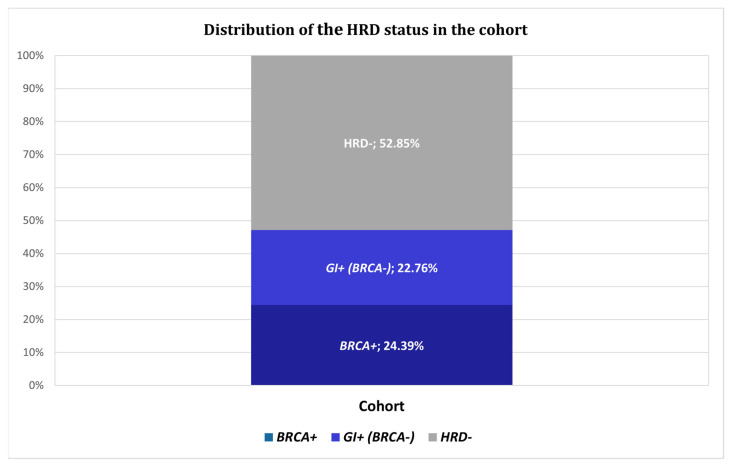
HRD status distribution among the 123 ovarian cancer patients with available *BRCA1/2* and GIS results.

When combining both markers, 58 patients (47.15%) were classified as homologous recombination deficient (HRD), defined as either *BRCA*-positive or GI-positive (Table 2, Figure 2). Notably, GI testing enabled the identification of a distinct subset of HRD-positive patients who were *BRCA*-wildtype, demonstrating the importance of GI incorporation beyond gene panel testing alone. The prevalence of HRD positivity in this ovarian cancer cohort is consistent with rates reported in high-grade serous ovarian carcinoma and underscores the clinical utility of integrating sWGS-based GI analysis with *BRCA* testing to optimize HRD detection [6,7].

Among patients identified as HRD-positive by sWGS analysis, follow-up data of 6–8 months were available for 20 individuals. Of these, 18 (90.0%) demonstrated sensitivity to platinum-based therapy. Sixteen patients received Olaparib treatment, with response data available for 12 cases. Among them, 5 (41.7%) achieved a complete response (CR), 3 (25.0%) a partial response (PR), 3 (25.00%) maintained stable disease (SD), and 1 (8.3%) experienced disease progression. The overall response rate (ORR; CR + PR) was 66.7% (95% CI: 39.1–86.2), while the disease control rate (DCR; CR + PR + SD) reached 83.3% (95% CI: 55.2–95.3). These findings provide preliminary evidence of the clinical value of sWGS-based HRD testing in guiding therapeutic decisions and predicting benefit from platinum-based and PARP inhibitor therapies, although the limited number of evaluable patients precludes any confirmatory conclusions.

### 2.2. Panel Analysis Results

**Figure 3:**
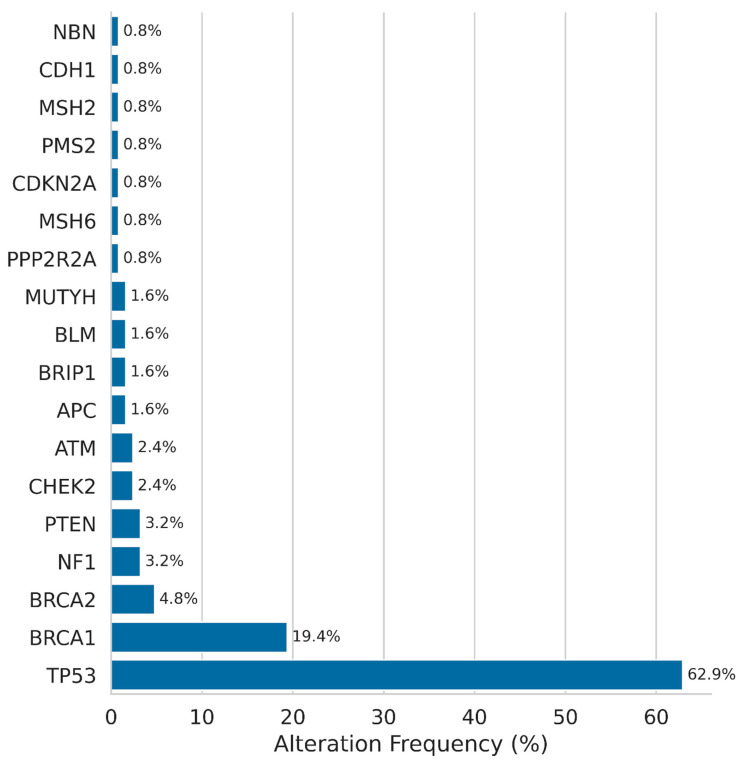
Prevalence of genes altered in the Ovarian Cancer Cohort.

At least one pathogenic alteration was present in 66.93% (83/124) of the patients tested. The most prevalent alteration was *TP53*, which was detected in 78 patients (62.90%). In 24 (19.35%) and 6 (4.83%) patients, respectively, pathogenic *BRCA1* and *BRCA2* variants were identified (Figure 3). Nine patients in the *BRCA1/2*-negative cohort exhibited pathogenic alterations in other genes of the HR pathway, including *ATM*, *CHEK2*, *BRIP1*, *RAD51C*, and *BARD1*. The majority of these cases (6/9) were classified as GI-low, while three were classified as GI-high. This finding implies that the functional impact of these variants on HRD is heterogeneous, as not all HR-related gene alterations result in increased genomic instability scores.

In addition to *TP53* and *BRCA1/2*, pathogenic or likely pathogenic gene alterations were also detected in *NF1* (3.23%), *PTEN* (3.23%), *CHEK2* (2.42%), and *ATM* (2.42%). In 1.61% of cases, alterations concerned *APC*, *BRIP1*, *BLM*, and *MUTYH* genes. Several other genes alterations were detected at lower frequency including *MSH6*, *CDKN2A*, *PMS2*, *MSH2*, *CDH1*, *PPP2R2A*, and *NBN*, present in 0.81% of the cases. These results are consistent with the well-established role of *TP53* and *BRCA1/2* alterations in high-grade serous ovarian carcinoma, while also highlighting the presence of rare potentially targetable alterations in other genes in a subset of patients.

In the *BRCA*-negative subgroup, *TP53* alterations were present in 58.6% (17/29) of patients with high GI and in 56.3% (36/64) of the GI-low patients. Statistical analysis using Fisher’s exact test revealed no significant association between *TP53* alteration status and GI classification (*p* = 1.0), indicating that *TP53* alterations occurred at a similar frequency irrespective of genomic instability status in this cohort. Due to the low alteration frequency, comparative analysis for other genes was not feasible in this cohort.

The importance of multigene analysis is evidenced by two notable results of MMR gene positive patients detected in our cohort. Both patients harbored pathogenic *BRCA2* variants at low allelic fractions (22–25%) together with high-allelic-fraction MMR gene alterations. Subsequent MSI testing revealed both cases to be MSI-high. Given the established tumor-agnostic efficacy of immune checkpoint inhibitors in MSI-high cancers, these patients would be candidates for anti-PD-1 therapy (e.g., pembrolizumab, nivolumab) according to current FDA and EMA guidelines. Although the presence of a *BRCA2* variant might support PARP inhibitor use, the predominant MMR-deficient phenotype likely represents the main driver of tumorigenesis in these cases.

## 3. Discussion

This study evaluated a shallow whole-genome sequencing (sWGS) approach for assessing genomic instability in ovarian cancer, using the Myriad myChoice test as the comparator. The strong agreement observed between the two methods, particularly for genomic instability status (GIS), indicates that the sWGS-based method can provide reliable HRD assessment in a clinical setting. Other groups have reported similar results using WGS approaches, supporting its potential as a cost-effective alternative to commercial HRD assays [13].

Previous studies indicated that approximately fifty percent of ovarian malignancies could be classified as HRD positive, and more than half of ovarian cancers might have been inadequately identified if solely BRCA status was assessed [9,17]. In the prospective ovarian cancer cohort, pathogenic/likely pathogenic *BRCA1/2* variants were detected in 24.19% of cases, with *BRCA1* alterations more frequent than *BRCA2*. This distribution is consistent with previous studies in high-grade serous ovarian carcinoma. Moreover, among the 93 patients without *BRCA1/2* alterations and evaluable GI assessment, an additional 22.76% were classified as HRD-positive based on GIS > 0.5. In total, 47.15% of the patients were classified as HRD positive based on either *BRCA1/2* status positivity or GI status. These findings support previous studies indicating that GI assessment complements *BRCA* testing and expands the identification of HRD-positive tumors [5,18]. In our cohort, the combined analysis of GI and *BRCA* status resulted in a twofold increase in the number of eligible patients when compared to *BRCA* analysis alone.

Given that the cost of the Myriad myChoice^®^ HRD test exceeds €3000, while the National Health Insurance reimbursement for such molecular analyses in Romania is approximately €1400, the implementation of a validated, locally available HRD testing solution—one that fits within current reimbursement frameworks—is essential to ensure equitable access to this clinically important biomarker. Furthermore, although reimbursement is available for BRCA1/2 analysis, genomic instability (GI) testing is only reimbursed for patients who are BRCA1/2-negative. This limitation further restricts access to comprehensive HRD assessment by centralized reference laboratories. Establishing an accredited, cost-effective local workflow—such as BRCA1/2 testing followed by GI analysis—would broaden patient eligibility, decrease diagnostic turnaround times, and facilitate the integration of HRD assessment into routine clinical practice.

In this study, sWGS-based local HRD testing effectively identified patients with a high likelihood of response to DNA damage–targeted therapies. Among HRD-positive cases, 90% demonstrated platinum sensitivity, and two-thirds achieved an objective response to PARP inhibitor therapy. Although the cohort size was limited, these preliminary findings are consistent with prior reports, including the PAOLA-1 trial and other translational studies, which have shown that HRD-positive tumors derive greater benefit from PARP inhibition [5,19].

Pathogenic variants in other homologous recombination (HR) pathway genes were identified in 9.57% of *BRCA1/2*-negative patients, affecting genes such as *ATM*, CHEK2 and BRIP1. Most of these patients (6/9) were GI-low, suggesting that in most cases non-*BRCA* HR gene alterations do not translate into the degree of genomic instability detected by this assay. In fact, most studies report a more prominent response to PARPi inhibitor treatment in *BRCA1/2* carriers, while evidence exists for other high risk genes such as *PALB2* and RAD51C, while low response rates and lower evidence exist for intermediate low risk genes such as CHECK2 and *ATM* [18,20]. This variability has been noted in various tumor types and is likely related to differences in gene function, redundancy in the HR pathway and the variable pathogenic impact of individual variants [18,20,21,22,23]. Functional HRD testing therefore remains important in addition to panel-based sequencing.

The overall mutation profile in our cohort reflected the high prevalence of *TP53* alterations characteristic of high-grade serous ovarian cancer. Although *TP53* status is not discriminatory within this histological subtype, it remains an important molecular hallmark and may have future therapeutic relevance as p53-targeting strategies advance [24].

Notably, two patients (IDs 13 and 118) harbored tumor *BRCA2* pathogenic variants at low VAF, alongside with pathogenic alterations in mismatch repair (MMR) genes, and both were MSI-high on subsequent analysis. For such cases, immunotherapy with PD-1 blockade may be a more appropriate option than PARP inhibition, given the robust evidence for immunotherapy efficacy in MSI-high tumors regardless of tissue origin [25,26]. Moreover, recent evidence supports the combination of PARPi and immune checkpoint inhibitors as a promising therapeutic option in various tumor types [27,28,29]. While most biomarker agnostic trials failed to prove benefit from such a combination strategy, early evidence highlights the promise of PARP–ICI combinations among genomically selected subgroups [29,30,31].

This study is to the best of our knowledge, the first to offer a comprehensive overview of genomic alterations and GI status in ovarian cancer patients from Romania, while also demonstrating the clinical value of HRD analysis. A major strength of our study is the inclusion of broad tumor molecular profiling, covering *BRCA1/2*, *TP53*, and other HR pathway genes, integrated with GI analysis for HRD assessment The cross platform genomic instability results comparison conducted contributes to ongoing international efforts aiming to harmonize HRD assays. Nevertheless, several limitations should be acknowledged. The comparatively limited number of study participants reduced the statistical power of the findings and the feasibility of conducting sub-group analyses. While the present validation sample size is small, the performance metrics and correlations support the technical robustness of the method. In addition, the availability of incomplete or missing clinical data limited the assessment of clinical response evaluation and may have introduced residual confounding. Extended follow-up collection is ongoing, and future studies will incorporate longer-term clinical outcomes. The study was also conducted within a single institutional context using a single platform, limiting the generalizability of the findings to other populations. Future prospective, multi-institutional studies will be essential for verifying and expanding our findings.

In conclusion, this first comprehensive analysis of Romanian ovarian cancer patients highlights the high prevalence of *TP53* and *BRCA1/2* alterations, as well as the distribution of genomic instability across histologic subtypes and clinical stages. The sWGS-based approach demonstrated high concordance with Myriad myChoice^®^ and may represent a feasible local alternative pending larger validation. Although larger prospective studies are necessary, our findings strongly support the incorporation of HRD testing into standard clinical practice in Romania.

## 4. Materials and Methods

### 4.1. Patient Cohort and Sample Collection

The study was conducted in accordance with the Declaration of Helsinki [32] and was approved by the Ethics Committee of the Department of Applied Genetics and Bio-technology, Faculty of Biology, University of Bucharest (Approval date: 10 September 2025, Approval code: BIO-ETH/60066/2025).

The study included three distinct sample sets. First, 24 archival ovarian cancer samples were used for assay validation; these had been previously characterized for *BRCA1/2* status and genomic instability. In addition, five commercially available reference materials with known *BRCA1/2* and HRD status were analyzed to assess assay accuracy and reproducibility. Specifically, Seraseq^®^ FFPE HRD (SeraCare, Milford, MA, USA) reference materials were used to evaluate the assay’s efficacy in HRD calculation: two positive samples (reference number 0710-2643) with expected GIS 72 ± 2, two low positive samples with reference 0710-2645 and expected GIS 54 ± 2 and two negative (0710-2644) with expected GIS 31 ± 2.

Moreover, the prospective cohort consisted of 124 consecutive patients with ovarian cancer who were referred by their treating physicians for *BRCA1/2* and HRD testing as part of routine clinical practice. Referral for testing was at the discretion of the clinician, and no predefined restrictions were imposed regarding histologic subtype or FIGO stage, although most cases represented newly diagnosed epithelial ovarian carcinomas (Supplementary Figure S1).

Inclusion criteria included pathology-confirmed ovarian cancer, availability of FFPE tumor tissue with ≥30% tumor content based on pathology review. Exclusion criteria included insufficient tumor material for DNA extraction, or inadequate DNA quality. One sample was excluded due to failure of GI analysis caused by low DNA integrity.

Clinical and histopathologic data were collected when available, including age at diagnosis, FIGO stage, and histologic subtype. Clinical response information was provided by the treating oncologists approximately 6–8 months after testing, resulting in evaluable follow-up data for 20 patients. Response to therapy was assessed according to RECIST v1.1 criteria [33].

### 4.2. DNA Extraction

DNA was extracted from FFPE tissue sections using the MagCore^®^ Genomic DNA FFPE One-Step kit (RBC Bioscience Co., Ltd., New Taipei City, Taiwan), following the manufacturer’s protocol. DNA quality and concentration were assessed to ensure suitability for downstream sequencing applications.

### 4.3. Multigene Next-Generation Sequencing (NGS) Analysis

Genomic DNA was analyzed using a custom target enrichment panel containing 52 genes, including *BRCA1*, *BRCA2*, and 21 genes of the homologous recombination repair (HR) pathway (KAPA HyperExplore Max 3Mb T1, NimbleGen, Roche, Basel, Switzerland). The panel also targeted clinically relevant regions from genes such as *TP53*, *ATM*, *CHEK2*, *RAD51C/D*, *PALB2*, *MSH2/6*, *PMS2*, *MLH1*, *POLE*, *POLD1*, *NF1*, *PTEN*, *APC*, among others [34].

Library preparation was performed using the Roche Kapa HyperPlus with Roche HyperCapture Custom Panel, and sequencing was carried out on the Aviti™ Next Generation Sequencing platform (Element Biosciences, San Diego, CA, USA). Reads were aligned to the human reference genome (GRCh37), and sequence variants were identified and interpreted with reference to a single clinically relevant transcript per gene. The assay covered all coding exons and 20 base pairs of flanking intronic regions (25 bp for *BRCA1/2*), with distinct target regions for *HOXB13*, *POLE*, and *POLD1*.

Sequencing depth was ≥500× across all targeted regions, with an effective mean depth >1000×, and >90% of bases achieving a Q30 quality score (99.9% base calling accuracy). Large genomic rearrangements (LGRs) were analyzed using the CE-IVD SeqPilot algorithm (JSI medical systems GmbH, Ettenheim, Germany) and confirmed by MLPA (MRC Holland, Amsterdam, The Netherlands). Pathogenic or likely pathogenic variants were classified according to ACMG/AMP guidelines, and Sanger sequencing was used for orthogonal confirmation of clinically significant findings.

### 4.4. Shallow Whole-Genome Sequencing (sWGS) and Genomic Instability Assessment

Low-coverage whole-genome sequencing (shallow WGS, sWGS) was performed to evaluate genomic instability status. Data were processed using the SeqOne automated bioinformatics pipeline [13,16]. The Genomic Instability Probability (GI Probability) was calculated based on two classes of features. The first class, Large Genomic Alterations (LGA), comprised copy number breakpoints between large genomic segments that satisfied predefined size and proximity thresholds, computed in multiple variant forms. The second class, Large-Scale Loss of Ploidy Segments (LPC), consisted of haploid genomic segments with lengths equal to or exceeding 10 Mb [16]. A machine learning model integrated multiple LGA and LPC metrics, applying a logit transformation to produce a probability score from 0 to 1. The HRD analysis model was originally developed and trained by the provider (SeqOne^®^, Montpellier, France) using a dataset of ovarian cancer samples from the PAOLA-1/ENGOT-OV25 phase III trial, for which Myriad myChoice GIS classifications were available as the reference [13]. Importantly, no recalibration or re-optimization of this threshold was performed in the present study to avoid overfitting. All samples were therefore classified according to the manufacturer-established cut-off (GI Probability > 0.5). A sample is classified as HRD-positive when the GIS probability exceeds 0.5, when a BRCA1/2 mutation is detected, or when both criteria are met.

### 4.5. Bottom of Form

#### Statistical Analysis

Categorical agreement between sWGS GI and Myriad GIS was assessed using the following metrics: sensitivity, specificity, overall percent agreement (OPA), positive predictive value (PPV), and negative predictive value (NPV). To enhance statistical transparency, all performance metrics—including sensitivity, specificity, positive predictive value (PPV), negative predictive value (NPV), and Cohen’s kappa—were accompanied by 95% confidence intervals (CIs), calculated using the Wilson score or exact binomial method, depending on the underlying distribution. Agreement between assays was measured using Cohen’s kappa coefficient (κ) to quantify inter-method reliability. Correlation between continuous GI scores obtained by the sWGS and Myriad assays was assessed using Pearson’s and Spearman’s correlation coefficients. For the PARP inhibitor subgroup, treatment efficacy was summarized using overall response rate (ORR) and disease control rate (DCR) with corresponding 95% confidence intervals calculated via the Wilson method. A two-tailed *p* value < 0.05 was considered statistically significant. Given the limited number of patients with clinical follow-up and treatment-response data, results from survival or outcome comparisons are presented without inferential statistics and are clearly labeled as exploratory. This approach ensures transparent reporting while maintaining appropriate interpretation consistent with the cohort size.

## Figures and Tables

**Table 1:** Concordance characteristics of Myriad MyChoice and the sWGS method for GI calculation.

Metric	Estimate	95% CI
Sensitivity	100.0% (14/14)	76.8–100
Specificity	90.0% (9/10)	55.5–99.7
PPV	93.3% (14/15)	68.0–99.8
NPV	100.0% (9/9)	66.4–100
Overall Percent Agreement	95.8% (23/24)	-
Cohen’s Kappa	0.913	0.72–1.00

**Table 2:** Combined *BRCA* and genomic instability status for HRD classification.

	HRD+	HRD−	Total
*BRCA1/2+*	30	0	30
*BRCA1/2-*	28	65	93
Total	58	65	123

## Data Availability

Data is contained within the article or Supplementary Material: The original contributions presented in this study are included in the article/Supplementary Material. Further inquiries can be directed to the corresponding author.

## Supplementary Materials

The following supporting information can be downloaded at: https://www.mdpi.com/article/10.3390/ijms262411968/s1.
