# Supplementary material for: Integrative sWGS: A New Paradigm for HRD Detection in Ovarian Cancer
Source: Int J Mol Sci. 2025 Dec 12;26(24):11968. doi: 10.3390/ijms262411968 (PMC12732861; doi:10.3390/ijms262411968)
Supplement: Supplementary file 1 [file ijms-26-11968-s001.zip › figureS1.pptx]

## Slide 1
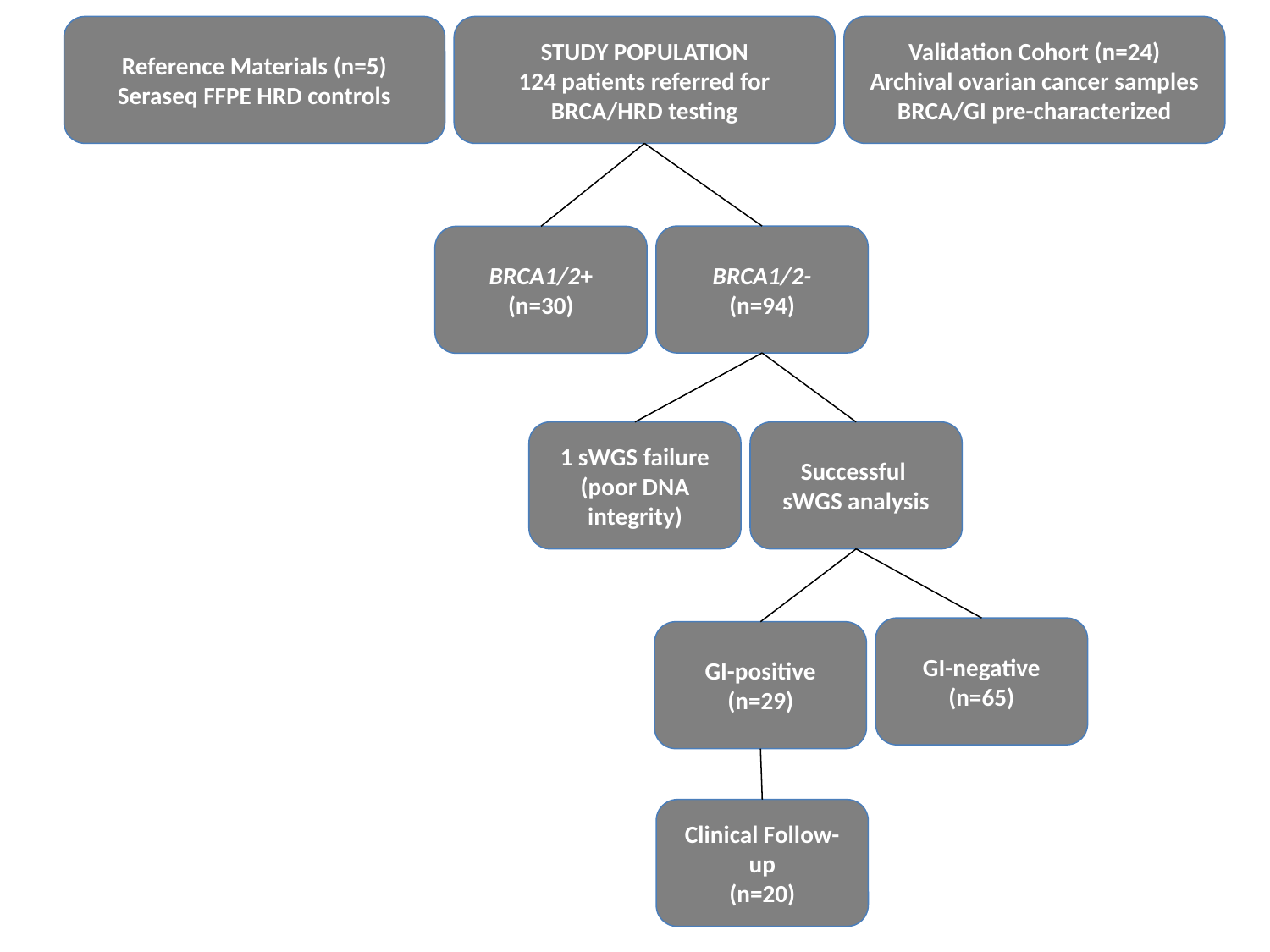

Reference Materials (n=5)
Seraseq FFPE HRD controls
STUDY POPULATION
124 patients referred for BRCA/HRD testing
Validation Cohort (n=24)
Archival ovarian cancer samples
BRCA/GI pre-characterized
BRCA1/2-
(n=94)
BRCA1/2+
(n=30)
1 sWGS failure (poor DNA integrity)
Successful sWGS analysis
GI-negative
(n=65)
GI-positive
(n=29)
Clinical Follow-up
(n=20)
